# Supplementary material for: Evaluating Back-to-Back and Day-to-Day Reproducibility of Cortical GABA+ Measurements Using Proton Magnetic Resonance Spectroscopy (1H MRS)
Source: Int J Mol Sci. 2023 Apr 23;24(9):7713. doi: 10.3390/ijms24097713 (PMC10178500; doi:10.3390/ijms24097713)
Supplement: Supplementary file 1 [file ijms-24-07713-s001.zip › Figure S2 supplemental.pdf]

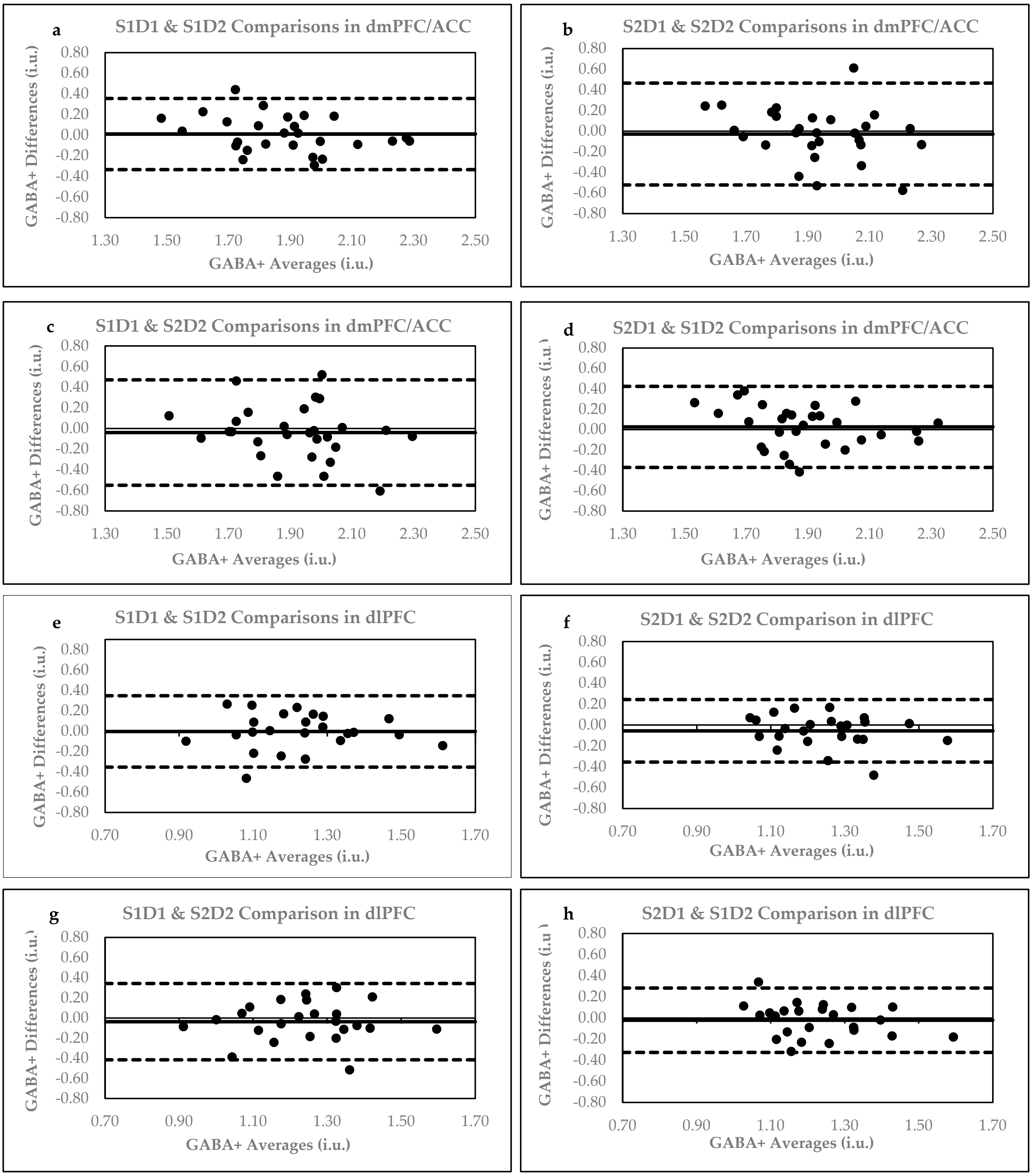

Figure S2 Supplemental. Bland-Altman plots for D2D comparisons of 5.2-number scans. a) S1D1 and S1D2 in dmPFC/ACC; b) S2D1 and S2D2 in dmPFC/ACC; c) S1D2 and S2D2 in dmPFC/ACC; d) S2D1 and S1D2 in dmPFC/ACC; e) S1D1 and S1D2 in dlPFC; f) S2D1 and S2D2 in dlPFC; g) S1D2 and S2D2 in dlPFC; h) S2D1 and S1D2 in dlPFC. S, scan; D, day; i.u., international units
