# Supplementary material for: Evaluating Back-to-Back and Day-to-Day Reproducibility of Cortical GABA+ Measurements Using Proton Magnetic Resonance Spectroscopy (1H MRS)
Source: Int J Mol Sci. 2023 Apr 23;24(9):7713. doi: 10.3390/ijms24097713 (PMC10178500; doi:10.3390/ijms24097713)
Supplement: Supplementary file 1 [file ijms-24-07713-s001.zip › Table S1 Supplemental.pdf]

Table S1 Supplemental. GABA+ concentrations and quality parameters in dmPFC/ACC. M, mean; SD, standard deviation; i.u., international units; FWHM, frequency width at half-maximum; SNR, signal-to-noise ratio; Hz, hertz; S, scan; D, day.

**Parameters M (SD)**	**S1D1** (n = 28)	**S2D1** (n = 28)	**S1D2** (n = 29)	**S2D2** (n = 29)	**Day 1 averages** (n = 28)	**Day 2 averages** (n = 28)
GABA+ (i.u.)	1.90 (0.20)	1.91 (0.19)	1.88 (0.24)	1.94 (0.24)	1.90 (0.17)	1.90 (0.22)
GABA+ FWHM (Hz)	20.98 (1.18)	21.02 (1.30)	21.04 (1.44)	21.22 (1.56)	21.00 (1.01)	21.03 (1.13)
H_20 FWHM (Hz)	10.92 (1.20)	10.76 (1.13)	11.09 (1.15)	10.90 (1.06)	10.84 (1.15)	10.93 (1.05)
GABA+ Fit Error%	4.71 (1.21)	4.99 (1.14)	4.86 (1.14)	4.99 (0.96)	4.85 (1.02)	4.91 (0.79)
H_20 Fit Error %	0.63 (0.09)	0.64 (0.08)	0.63 (0.08)	0.64 (0.07)	0.63 (0.08)	0.63 (0.07)
GABA+ SNR	13.95 (2.94)	14.04 (2.84)	13.62 (2.16)	13.82 (2.04)	14.00 (2.64)	13.73 (1.85)
H_20 SNR	18344.82 (5893.39)	18709.42 (6815.02)	17716.73 (4776.56)	17980.87 (5080.79)	18527.12 (6237.01)	17481.71 (4280.53)
