# Supplementary material for: Evaluating Back-to-Back and Day-to-Day Reproducibility of Cortical GABA+ Measurements Using Proton Magnetic Resonance Spectroscopy (1H MRS)
Source: Int J Mol Sci. 2023 Apr 23;24(9):7713. doi: 10.3390/ijms24097713 (PMC10178500; doi:10.3390/ijms24097713)
Supplement: Supplementary file 1 [file ijms-24-07713-s001.zip › Table S2 Supplemental.pdf]

Table S2 Supplemental. GABA+ concentrations and quality parameters in dlPFC. M, mean; SD, standard deviation; FWHM, i.u.; international units; frequency width at half-maximum; SNR, signal-to-noise ratio; Hz, hertz; S, scan; D, day.

**Parameters M (SD)**	**S1D1** (n = 24)	**S2D1** (n = 24)	**S1D2** (n = 25)	**S2D2** (n = 25)	**Day 1 Averages** (n = 23)	**Day 2 Averages** (n = 23)
GABA+ (i.u.)	1.24 (0.17)	1.22 (0.14)	1.23 (0.18)	1.27 (0.18)	1.23 (0.15)	1.26 (0.15)
GABA+ FWHM (Hz)	20.55 (1.72)	20.50 (1.54)	20.19 (1.53)	20.40 (1.72)	20.46 (1.29)	20.36 (1.25)
H_20 FWHM (Hz)	12.16 (1.36)	1.91 (1.08)	12.25 (1.22)	11.66 (0.99)	11.99 (1.03)	11.70 (1.05)
GABA+ Fit Error%	5.15 (1.45)	5.21 (1.15)	5.37 (1.51)	5.23 (0.93)	5.09 (1.03)	5.30 (1.02)
H_20 Fit Error %	0.73 (0.13)	0.69 (0.11)	0.73 (0.15)	0.71 (0.13)	0.71 (0.11)	0.72 (0.13)
GABA+ SNR	13.85 (2.62)	13.03 (1.94)	13.53 (2.11)	13.24 (1.82)	13.36 (2.14)	13.42 (1.78)
H_20 SNR	15047.37 (5491.52)	14982.63 (5105.4)	13539.57 (4754.51)	14625.55 (5487.87)	15215.34 (5213.69)	14031.84 (5068.51)
